# The Lancaster Sensorimotor Norms: multidimensional measures of perceptual and action strength for 40,000 English words

**DOI:** 10.3758/s13428-019-01316-z

**Published:** 2019-12-12

**Authors:** Dermot Lynott, Louise Connell, Marc Brysbaert, James Brand, James Carney

**Affiliations:** 1 Department of Psychology, Lancaster University, Lancaster, UK; 2 Department of Experimental Psychology, Ghent University, Ghent, Belgium; 3 New Zealand Institute of Language Brain and Behaviour, University of Canterbury, Canterbury, New Zealand; 4 Department of Arts and Humanities, Brunel University London, London, UK

## Abstract

Sensorimotor information plays a fundamental role in cognition. However, the existing materials that measure the sensorimotor basis of word meanings and concepts have been restricted in terms of their sample size and breadth of sensorimotor experience. Here we present norms of sensorimotor strength for 39,707 concepts across six perceptual modalities (touch, hearing, smell, taste, vision, and interoception) and five action effectors (mouth/throat, hand/arm, foot/leg, head excluding mouth/throat, and torso), gathered from a total of 3,500 individual participants using Amazon’s Mechanical Turk platform. The Lancaster Sensorimotor Norms are unique and innovative in a number of respects: They represent the largest-ever set of semantic norms for English, at 40,000 words × 11 dimensions (plus several informative cross-dimensional variables), they extend perceptual strength norming to the new modality of interoception, and they include the first norming of action strength across separate bodily effectors. In the first study, we describe the data collection procedures, provide summary descriptives of the dataset, and interpret the relations observed between sensorimotor dimensions. We then report two further studies, in which we (1) extracted an optimal single-variable composite of the 11-dimension sensorimotor profile (Minkowski 3 strength) and (2) demonstrated the utility of both perceptual and action strength in facilitating lexical decision times and accuracy in two separate datasets. These norms provide a valuable resource to researchers in diverse areas, including psycholinguistics, grounded cognition, cognitive semantics, knowledge representation, machine learning, and big-data approaches to the analysis of language and conceptual representations. The data are accessible via the Open Science Framework (http://osf.io/7emr6/) and an interactive web application (https://www.lancaster.ac.uk/psychology/lsnorms/).

My whole body remembered it, the familiar scrape of stone against my palm, the brace of my thigh muscle as I pushed myself upwards, into the whirl of green and exploding light. — Tana French 2007, *In the Woods*, p. 589

Sensorimotor information is central to how we experience and navigate the world. We acquire information through our senses, while our bodies provide feedback, as we physically interact with objects, people, and the wider environment. Many theoretical views of cognition describe a fundamental role for such sensorimotor knowledge in conceptual thought (e.g., Barsalou, 1999; Connell, 2019; Connell & Lynott, 2014b; Smith & Gasser, 2005; Vigliocco, Meteyard, Andrews, & Kousta, 2009; Wilson, 2002), with numerous empirical demonstrations supporting such claims (e.g., Connell, Lynott, & Dreyer, 2012; Kaschak, Zwaan, Aveyard, & Yaxley, 2006; Matlock, 2004; Zwaan & Taylor, 2006).

To test such embodied (or grounded) theories of cognition, researchers need appropriate stimuli for empirical tests and for developing mathematical or computational models. Lynott and Connell (2009, 2013) developed a set of modality-specific sensory norms for concepts for which each sensory modality (e.g., auditory, gustatory, haptic, olfactory, or visual) maps onto distinct cortical regions (e.g., gustatory cortex, auditory cortex, etc.). By having individuals provide ratings for each modality separately, the norms capture the extent to which something is experienced across different sensory modalities, without risk of ignoring or distorting the role of particular modalities (Connell & Lynott, 2012a, 2016a). Subsequent empirical studies have shown that such modality-specific measures are good predictors of people’s performance across a range of cognitive tasks (e.g., lexical decision, word naming, and property verification), and often outperform long-established measures such as concreteness and imageability (e.g., Connell & Lynott, 2012a, 2014a). For example, in examining performance on lexical decision and word-naming (reading-aloud) tasks, Connell and Lynott (2012a) found that modality-specific experience (specifically, the extent of perceptual experience in the strongest modality for a given concept, its “maximum perceptual strength”) was a more reliable predictor of performance than both concreteness and imageability. Closer analysis showed that concreteness ratings appeared to capture separate decision criteria, and some modality-specific information was either ignored or skewed by raters. Moreover, imageability ratings were heavily biased toward the visual modality at the expense of other modalities. Connell and Lynott (2016a) also found that when people are asked to rate sensory experience generally (as opposed to focusing on one modality at a time), it can lead to extensive loss of information (e.g., modality-specific auditory, gustatory, and haptic information was neglected by people, whereas information from olfactory and visual dimensions was distorted). Critically, this information loss was reflected in weaker semantic facilitation in word recognition, a phenomenon that results from automatic and implicit access to the grounded representation of a word’s meaning: maximum perceptual strength derived from modality-specific measures outperformed overall sensory experience ratings (Juhasz & Yap, 2013) in predicting latency and accuracy of lexical decision judgments. These findings highlight the importance of individually rating separate perceptual modalities when norming the sensory basis of words and concepts.

An added advantage of using modality-specific measures of sensory experience is that they allow researchers to tap into effects that relate to particular modalities and not others. Connell and Lynott (2010) showed how a processing disadvantage for tactile stimuli observed during perceptual processing was also observed when processing modality-specific words. That is, in perception, people are poorer at detecting tactile stimuli relative to auditory and visual stimuli (Spence, Nicholls, & Driver, 2001). Using a signal detection paradigm, Connell and Lynott (2010) found that people were similarly poorer at detecting tactile-related words (e.g., *sticky*) relative to other modalities. Connell and Lynott (2014a) also derived contrasting modality-specific predictions relating to lexical decision and reading aloud for individual words. Because lexical decision is a primarily visual task, it necessarily focuses attention on the visual system, and preallocation of attention to vision facilitates processing of semantic information related to the visual modality (e.g., Connell et al., 2012; Foxe, Simpson, Ahlfors, & Saron, 2005). Thus, for lexical decisions, Connell and Lynott (2012b, 2014a) found that strength of perceptual experience in the visual modality (but not the auditory modality) was a reliable predictor of performance in that task. By contrast, reading aloud also requires attention on the auditory modality, as participants must plan and monitor their speech output to ensure correctly articulated responses. Consistent with this idea, strength of auditory experience and strength of visual experience were *both* reliable predictors for performance for the reading aloud task. Such phenomena could not have been detected using extant measures (e.g., concreteness or imageability ratings) because they do not offer sufficient granularity in terms of perceptual experience. Thus, modality-specific measures of sensory experience provide the capacity to generate and test novel predictions related to modality-specific processing and representations.

The above work on perceptual strength concentrated on five common modalities of sensory experience: touch, hearing, smell, taste, and vision. More recently, however, Connell, Lynott, and Banks (2018) showed that interoception (i.e., sensations inside the body) also plays an important role in semantic representations, and could be a primary grounding mechanism for abstract concepts. As compared to more concrete concepts like *banjo* or *rainy*, strength of interoceptive experience was higher for abstract concepts like *hungry* and *serenity*, and was particularly important for emotion concepts such as *fear* and *happiness*. Moreover, including interoceptive strength in a measure of maximum perceptual strength enhanced semantic facilitation in lexical decision performance (as compared to the original set of five modalities). Overall, modality-specific measures of interoceptive experience seem to be just as important as measures from other perceptual modalities in capturing the sensory basis of semantic knowledge.

Since the original appearance of modality-specific norms of perceptual strength, interest in their broad utility has led other research groups to extend them in a variety of directions. Perceptual strength norms (also termed modality exclusivity norms, after the original Lynott & Connell, 2009, work) now exist in several different languages, including Russian (Miklashevsky, 2018), Serbian (Filipović Đurđević, Popović Stijačić, & Karapandžić, 2016), Dutch (Speed & Majid, 2017), and Mandarin (Chen, Zhao, Long, Lu, & Huang, 2019), and have been developed for concept–property pairs as well as individual words (van Dantzig, Cowell, Zeelenberg, & Pecher, 2011). The original English-language norms have also been applied in novel ways, such as examining stylistic differences of authors (Kernot, Bossomaier, & Bradbury, 2019), studying perceptual metaphors (e.g., *rough sound*, *smooth melody*; Winter, 2019), testing models of lexical representations (Johns & Jones, 2012), evaluating the iconicity of words in written (Winter, Perlman, Perry, & Lupyan, 2017), and signed languages (Perlman, Little, Thompson, & Thompson, 2018), and discovering links between sensory and emotional experience (Winter, 2016).

Nonetheless, a notable gap in the work discussed above is that it focuses solely on sensory experience, and has not included parallel measures of action or effector-specific experience. However, there is good evidence for the relevance of action experience to people’s semantic representations of concepts (e.g., Glenberg & Gallese, 2012; Hauk, Johnsrude, & Pulvermüller, 2004). For instance, manual action verbs such as *throw* activate some of the same motor circuits as moving the hand (Hauk et al., 2004), and their processing is selectively impaired in patients with Parkinson’s disease, which entails neurodegeneration of the motor system (Boulenger, Hauk, & Pulvermüller, 2008; Fernandino et al., 2013). Critically, the motor basis to semantic knowledge is specific to the bodily effector used to carry out a particular action. Applying transcranial magnetic stimulation (TMS) to hand and leg areas of the motor cortex differentially influences processing of hand and leg action words: Hand area TMS facilitates lexical decision of hand action words, such as *pick*, relative to leg action words, such as *kick*, whereas this effect is reversed with leg area TMS (Pulvermüller, Hauk, Nikulin, & Ilmoniemi, 2005; see also Klepp et al., 2017). Such double dissociations in motor-language facilitation underscore the importance of individually examining separate action effectors when norming the motor basis of words and concepts.

Some existing measures have attempted to capture action knowledge, but have alternatively used feature production tasks, as opposed to rating dimensions of action (i.e., in which people verbally list the features associated with concepts: McRae, Cree, Seidenberg, & McNorgan, 2005; Vinson & Vigliocco, 2008), focused on generalized action (e.g., body–object interaction: Tillotson, Siakaluk, & Pexman, 2008; relative embodiment: Sidhu, Kwan, Pexman, & Siakaluk, 2014; see Connell & Lynott, 2016b, for review), or focused on a restricted subset of action types (e.g., graspability: Amsel, Urbach, & Kutas, 2012; actions associated with lower limb, upper limb, or head: Binder et al., 2016) that omits other parts of the body involved in action. For example, the action of *pushing* can also involve the torso (Moody & Gennari, 2010), and mouth actions are cortically distinct from other actions of the face (Meier, Aflalo, Kastner, & Graziano, 2008). To our knowledge, therefore, no large-scale set of norms taps into a comprehensive range of effector-specific action experience. In the present work, we address this gap by collecting effector-specific action strength norms for a large number of concepts.

Overall, there is good evidence for the internal reliability, face validity, and predictive value of existing modality-specific measures of experience, but studies to date have been hampered by their relatively small scale (typically a few hundred items). Because megastudies with tens of thousands of words are increasingly used across the cognitive sciences to enable greater statistical power, reduce experimenter bias in item selection, and increase study reliability (e.g., Balota, Yap, Hutchison, & Cortese, 2012; Brysbaert, Mandera, & Keuleers, 2018; Kuperman, Estes, Brysbaert, & Warriner, 2014), it has become essential to provide suitably large sets of norms. In the present study, we present sensorimotor norms across 11 dimensions for approximately 40,000 concepts, comprising six modality-specific dimensions of perceptual strength (auditory, gustatory, haptic, olfactory, visual, interoceptive) and five effector-specific dimensions of action strength (head, arm/hand, mouth, leg/foot, torso). Because the modality-specific measures of perceptual strength map to specific, separable regions of the somatosensory and insular cortex (e.g., Craig, 2003; Goldberg, Perfetti, & Schneider, 2006), so too do the effector-specific measures of action strength map to specific, separable regions of the motor cortex (e.g., Aflalo & Graziano, 2006).

To the best of our knowledge, these norms represent the largest ever set of semantic norms for English, and bring the following benefits. First, incorporating almost 40,000 words, they provide far greater lexical coverage than has been possible with previous norms, encompassing the majority of words known to an average adult speaker of English (i.e., approximating a full-size adult conceptual system; Brysbaert, Warriner, & Kuperman, 2014). Second, they extend existing norms to new sensory modalities (i.e., interoception), which have been found to be especially important for how people represent emotion-related concepts (Connell et al., 2018). Third, the norms go beyond perceptual strength to include action strength with a range of effectors that spans the full body, allowing researchers to consider a far greater range of object-related experiences, and to test effector-specific theoretical predictions. Fourth, with approximately 40,000 words × 11 dimensions (plus cross-dimensional variables), the scale of the norms also provides the statistical power for a wide range of applications, including examining complex relations between variables that provide sensorimotor grounding for concepts; identifying subtle, but potentially important, semantic effects and interactions in language processing; and using the norms in machine learning techniques such as document categorization.

## Study 1: Sensorimotor norming

### Method

From the main Open Science Foundation (OSF) project page for this project (https://osf.io/7emr6/), readers can access all materials and data; scripts for experiments and data analysis; and full results of analysis. Researchers can also access item-level norms via a web interface, located at https://www.lancaster.ac.uk/psychology/lsnorms/. In addition to the aggregated data, we also separately include full trial-level data from participants, also downloadable from the project’s OSF page.

***Materials*** Our original item set was a total of 39,954 English lemmas, comprising 37,058 single-word items (e.g., *bus*) and 2,896 lexicalized two-word items (e.g., *bus stop*). All items were taken from Brysbaert, Warriner, and Kuperman’s (2014) work on concreteness ratings, which represented all lemmas known by at least 85% of native speakers of English, and used American English spellings.^1^ The dataset contains words from major syntactic categories (e.g., nouns, verbs, prepositions, pronouns, adjectives, adverbs, etc.) and a wide range of concepts (e.g., foods, animals, emotions, sports, taboo words, professions, colors, etc.) We divided the total item set into 821 lists of 48 test items plus a constant set of five calibrator and five control words that appeared in all lists (see below). Thus, each list rated by participants consisted of 58 items. Following initial testing, 20 additional lists were created that had a different number of items (up to a maximum of 64 items) to include words that received only a low number of valid ratings when first tested, and some words that were missing from the original master list of words. All lists were created using the same criteria described below.

Five calibrator words were presented to participants at the start of each item list, in the same order, to introduce participants to unambiguous examples of items that spanned the full range of sensorimotor strength in different dimensions. We selected the calibrator words separately for perceptual and action strength norming from items that were 100% known in Brysbaert et al.’s (2014) concreteness norms. For perceptual strength norming, the calibrator words were taken from existing norms (Lynott & Connell, 2009, 2013, plus other unpublished ratings available here: https://www.lancaster.ac.uk/staff/connelll/lab/norms.html) to have low variance in ratings across participants and to provide examples of the following criteria (calibrator word given in brackets): low strength across all modalities (*account*), medium strength across multiple modalities (*breath*), high strength in a single modality (*echo*), uneven strength across modalities (*hungry*), and high strength across multiple modalities (*liquid*). For motor strength norming, the calibrator words were selected according to ratings from a pilot study (*N* = 20 native speakers of English from Lancaster University who did not take part in any other norming tasks) to have low variance in ratings across participants and to provide examples of the following criteria: low strength across all effectors (*shell*), medium strength across multiple effectors (*tourism*), high strength across multiple effectors (*driving*, *breathe*), and high strength in a single effector (*listen*).

In addition, five controls words were randomly interspersed throughout the item list in order to provide a means to evaluate participant performance as part of our data quality checks (see the Data Quality and Exclusions section). We selected control words using the same criteria as for calibrator words. For perceptual strength norming, the control words were *grass*, *honey*, *laughing*, *noisy*, and *republic*; for action strength norming, the control words were *bite*, *enduring*, *moving*, *stare*, and *vintage*.

To populate the item lists, we used data binning to ensure each list contained items that varied both by their likelihood of being known to participants and by concreteness. We first performed a quartile split over all items according to the percentage of respondents in Brysbaert et al.’s (2014) study who knew the word, giving four bins with percentage known in the intervals 85.0%–93.1%, 93.1%–100%, 100%–100%, and 100%–100%. Since over half the items were known by all participants (i.e., 100% known), the last two quartiles, and some of the second quartile, were undifferentiated by percentage known: we therefore used random sampling to allocate 100% known items to their relevant bins. We then performed a second quartile split on each of these four bins based on Brysbaert et al.’s concreteness ratings, which meant that each percentage-known bin was further divided into four bins of most to least concrete, producing in 16 bins in total. Finally, in order to create each item list of 48 items, we drew a random sample of three items without replacement from each of the 16 bins.

#### Participants

A total of 3,500 unique participants completed 32,456 surveys via Amazon’s Mechanical Turk platform (*M* = 7.12 item lists per participant). Participants could complete more than one word list, but could not complete the same list twice. Ratings for perceptual strength and action strength were gathered separately. That is, for a given list of words, a participant rated either all modalities of perceptual strength or all effectors of action strength but not both at the same time (although it was possible for individual participants to contribute to both perceptual and action strength norms on different occasions). The perceptual strength norms component of the study had 2,625 participants, with participants completing on average 5.99 lists each; the action strength norms component had 1,933 participants, with participants completing an average of 8.67 lists each. The participants were self-selected and had English as their first language. We recruited only experienced MTurk users who had already completed over 100 tasks (i.e., MTurk HITS > 100) with high-quality performance (i.e., > 97% HIT approval). We used TurkPrime (an interface to Mturk: Litman, Robinson, & Abberbock, 2016) to block duplicate IP addresses from completing the same item list, and to microbatch data collection into small groups of participants (which reduced overall costs and ensured the HITs were advertised over a longer time window). Participants were remunerated at US$2.75 per completed perceptual strength item list and US$2.25 per completed action strength item list (i.e., intended to reflect payment at US$9 per hour, pro rata, according to pilot timings of the task). Table 1 summarizes participant characteristics for age and sex. Ethics approval for the project was granted by Lancaster University Research Ethics Committee.

**Table 1:** Participant demographics for the separate components of data collection in the sensorimotor norms

Component	Sex	*N*	Age (Mean)	Age (*SD*)
Perceptual strength	Female	1,230	36.9	11.0
	Male	1,371	33.3	9.4
	Prefer not to say	4	37.0	21.4
	Missing	20		
	*All*	*2,625*	*35.0*	*10.4*
Action strength	Female	927	37.6	10.7
	Male	1,000	34.0	9.8
	Prefer not to say	4	35.0	22.7
	Missing	2		
	*All*	*1,933*	*35.7*	*10.4*
Sensorimotor combined	Female	*1,644*	*36.8*	*10.8*
	Male	*1,823*	*33.2*	*9.5*
	Prefer not to say	*12*	*30.2*	*12.8*
	Missing	*21*		
	*All*	*3,500*	*34.9*	*10.3*

Numbers are also provided for the overall combined dataset (in italics; providing the total number of unique participants), as some participants provided ratings for both the perceptual and action strength norms.

#### Data collection procedure

**Fig. 1:**
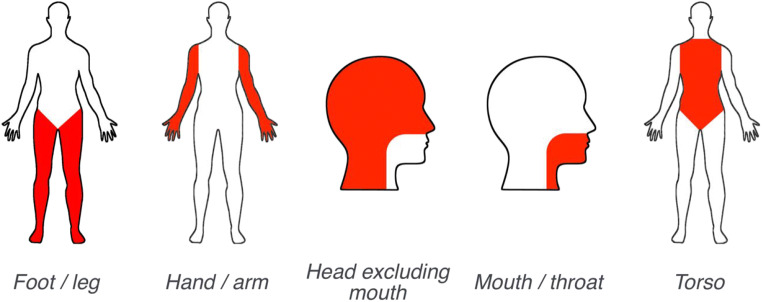
Avatar images used to describe the area of each effector during action strength norming.

Using Qualtrics survey software, we created a template survey that followed procedures developed for the original perceptual strength norms of Lynott and Connell (2009) and Lynott and Connell (2013). At the start of the survey, participants read an information sheet and indicated their informed consent to continue with the study. Participants then saw a detailed instructions screen that explained they would be asked to rate how much they experience everyday concepts using six perceptual senses/five action effectors from different parts of the body. The instructions further explained that there were no right or wrong answers and participants should use their own judgment; that the rating scales ran from 0 (*not experienced at all with that sense/action*) to 5 (*experienced greatly with that sense/action*); and that if participants did not know the meaning of a word, they should check the “Don’t know the meaning of this word” box and move onto the next item. Participants also received a warning that because the study used words encountered in everyday life, this would occasionally mean that some words might be offensive or explicit, and participants were reminded of their right to withdraw. To aid participants in discriminating between the five different effectors during action strength norming, we presented images of a human avatar, to highlight the body part indicated by each effector (see Fig. 1).

Each item appeared individually on its own rating screen in a short piece of framing text followed by the relevant rating scales. For perceptual strength norming, the text read “To what extent do you experience WORD,” where WORD was replaced with the item in question in uppercase text. Underneath were six rating scales, one for each of the perceptual modalities under investigation, labeled “By feeling through touch,” “By hearing,” “By sensations inside your body,” “By smelling,” and “By tasting”; the order of these modalities was randomized for each item list. For action strength norming, the text read “To what extent do you experience WORD by performing an action with the,” where WORD was replaced with the item in question in uppercase text. Underneath were five rating scales, one for each action effector, labeled “Foot/leg,” “Hand/arm,” “Head excluding mouth,” “Mouth/throat,” and “Torso”; the order of these effectors was randomized for each item list. The label of each action effector was accompanied by a small version of the body avatar that was presented in the instructions. No default value was selected on any rating scale. At the bottom of the screen were a check box labeled “Don’t know the meaning of this word” and a button labeled “Next.” Example images of the rating screens can be accessed on the OSF project page. Participants could only move onto the next item if they had either (a) selected a rating on all scales or (b) checked the “Don’t know” box. Participants were free to change their ratings until they clicked the “Next” button, but it was not possible to return to previous items once they had moved on.

Each list of 58 items started with the calibrator words, followed by the 48 test items and five control items in random order. The study was self-paced and was timed in piloting to be completed in approximately 18 min, for perceptual strength norming, and 15 min, for action strength norming.

#### Data checks and exclusions

To ensure the data collected were of sufficiently high quality, we instituted a number of checks based on individual (participant) performance, item completion, and inter-rater agreement per list of words.

Every completed data file (i.e., the responses of a particular participant to a particular item list) was checked individually. A data file was excluded as poor-quality data if the participant responded “Don’t know the meaning of this word” to one or more of the control words (all of which were known by 100% of participants in the Brysbaert et al., 2014, concreteness norms), or if their ratings for the control words correlated at *r* < .2 with the existing norms for those words (see the Materials section); in all, 72 data files were excluded on this basis. Additionally, a small number of participants completed the same item list more than once due to incomplete screening criteria in early testing; when this happened, we retained only the first-submitted data file per participant and excluded the duplicates (27 in total). Words were considered as valid if they had at least ten ratings each for both perceptual and action ratings. In total 247 words were lost due to having low numbers of valid ratings. Note that we are making available the norms for these “low-*N*” words in a separate file from the main norms, as researchers may find them suitable for some purposes (e.g., where the focus is on sensory modalities and there are a sufficient number of ratings from the perceptual norms). We used imputation to resolve the issue of missing values for those items with a low number of responses. This was done for all lists, using multivariate imputation by chained equations (MICE; van Buuren & Groothuis-Oudshoorn, 2011), and the final reported norms do not include any imputed values. From the remaining high-quality data files, we calculated Cronbach’s (1951) alpha per item list per dimension: Responses were retained when the mean alpha across all dimensions was ≥ .8 and each individual dimension had alpha ≥ .7 (i.e., very good agreement overall).

**Fig. 2:**
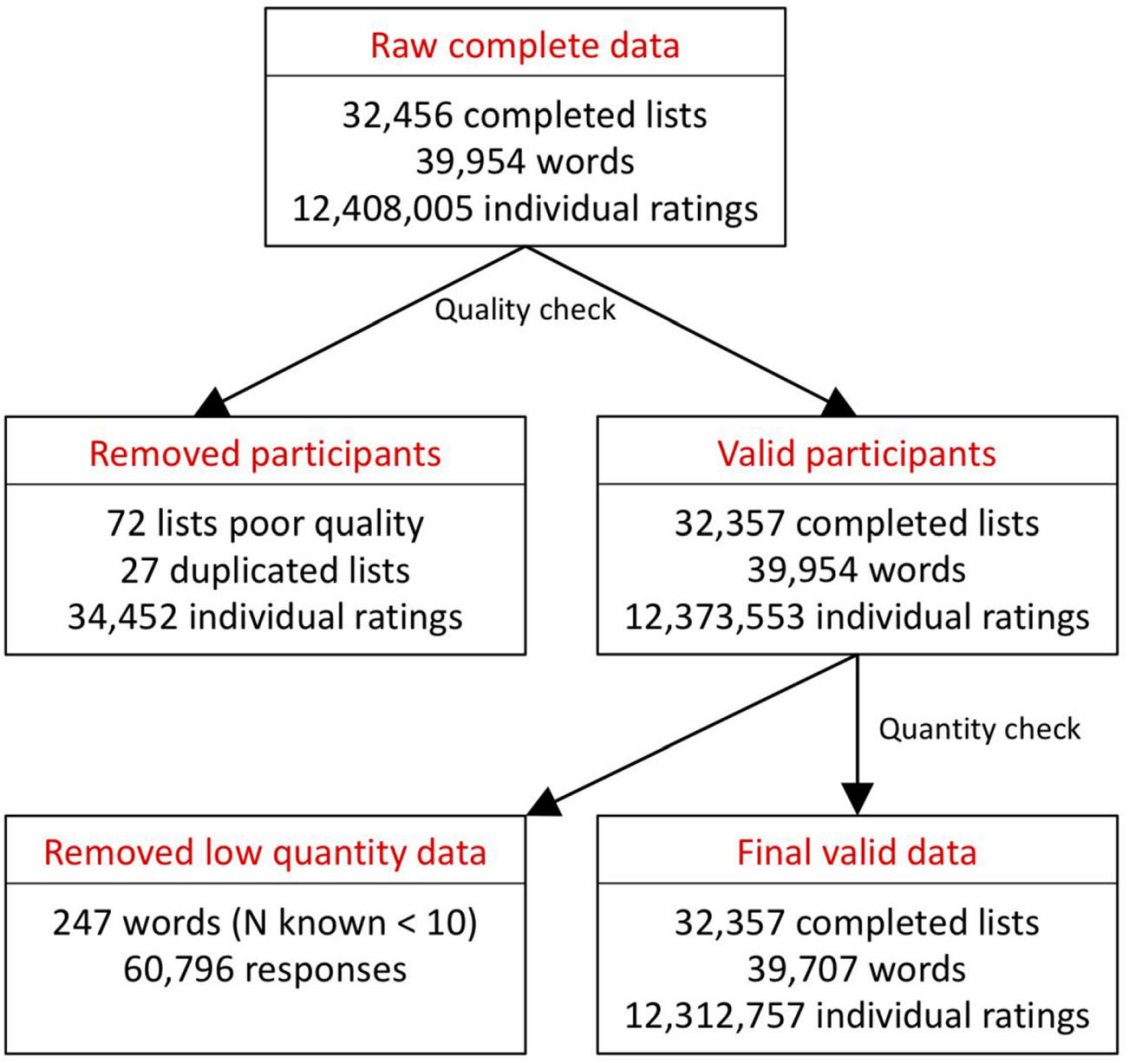
Schematic of data exclusions at each stage of the data preparation process

Figure 2 summarizes data loss due to various exclusion criteria: Overall, a very low proportion of the data had to be excluded (0.6% of words, 0.8% of individual ratings). Our final item set thus comprised 39,707 lemmas, each of which had valid norming data in 11 sensorimotor dimensions. On average, the sensorimotor strength ratings for each item were based on 18.0 participants in the perceptual component (range *N* = [10, 74]), and 19.1 participants in the action component (range *N* = [10, 58]). These means exclude the control and calibrator words, which appeared in every item list and were therefore rated by almost all participants (means: perceptual *N* = 15,948.4, action *N* = 16,659.2).

### Results and discussion

**Fig. 3:**
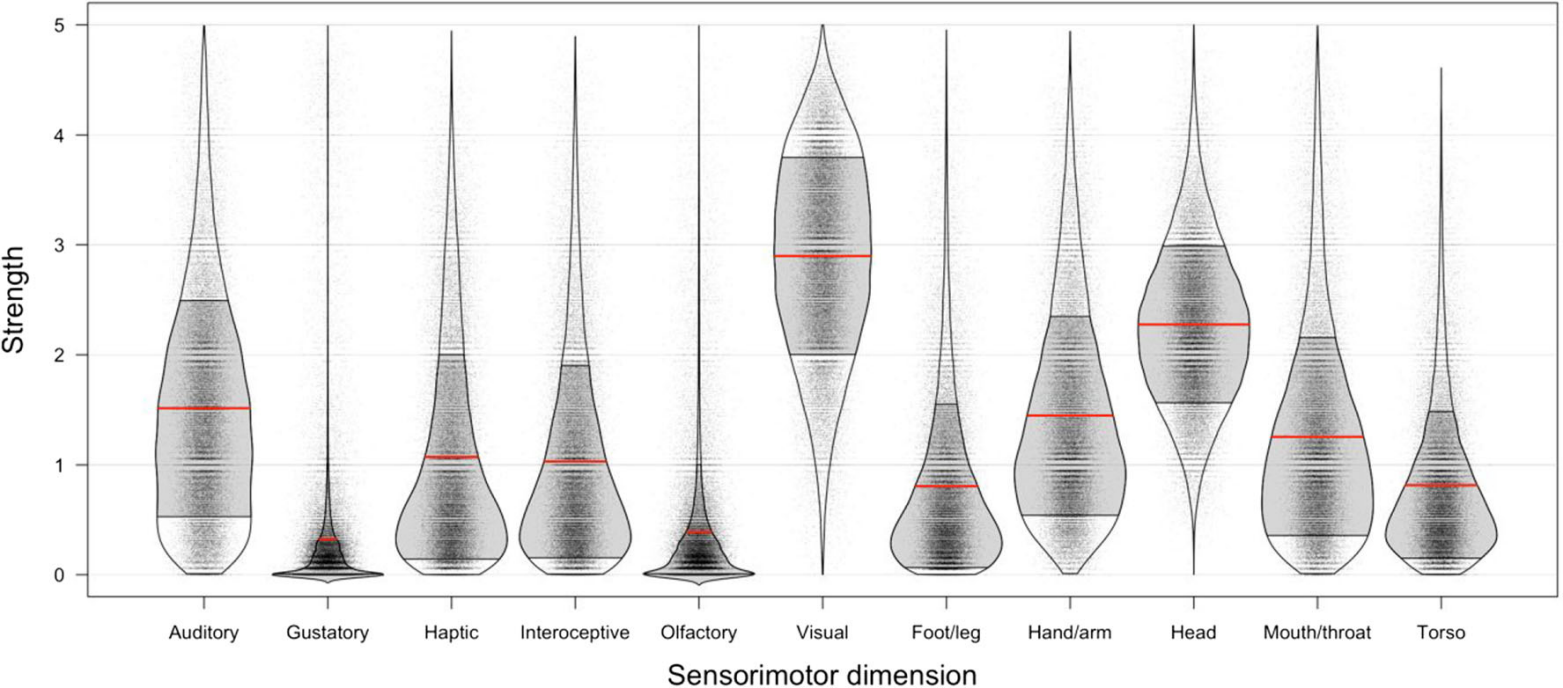
Violin plots showing the distribution of sensorimotor strength ratings per dimension and level of spread. Dots indicate the data points for individual items (*N* = 39,707), solid red lines indicate mean ratings, and the shaded areas indicate ± 1 *SD*

We calculated summary statistics (mean rating and standard deviations) per dimension per word. Table 2 and Fig. 3 (violin plots) show the mean ratings per perceptual modality and action effectors, along with measures of spread. The interrater reliability was excellent for all dimensions: The mean Cronbach’s alpha (calculated per item list and then averaged) for each perceptual modality (auditory .93, gustatory .96, haptic .92, interoceptive .92, olfactory .94, visual .90) was comparable to that from previous perceptual strength norms that had been collected in a traditional lab setting (Lynott & Connell, 2013), and the alpha values for action effectors were similar (foot .93, hand .91, head .85, mouth .92, and torso .89).

**Table 2:** Mean sensorimotor strength ratings (0–5), standard deviations (*SD*), standard errors (*SE*), and uniqueness scores (proportions of unique variance, 0–1) per sensorimotor dimension for the full sensorimotor norms of 39,707 words

Sensorimotor Dimension	*M*	*SD*	*SE*	Uniqueness
*Perceptual Modality*				
Auditory	1.51	0.99	0.005	.349
Gustatory	0.32	0.70	0.003	.122
Haptic	1.07	0.93	0.005	.319
Interoceptive	1.03	0.88	0.004	.342
Olfactory	0.39	0.62	0.003	.253
Visual	2.90	0.90	0.005	.267
*Action Effector*				
Foot/leg	0.81	0.75	0.004	.282
Hand/arm	1.45	0.91	0.005	.306
Head	2.28	0.72	0.004	.431
Mouth/throat	1.26	0.90	0.005	.281
Torso	0.82	0.67	0.003	.187

**Fig. 4:**
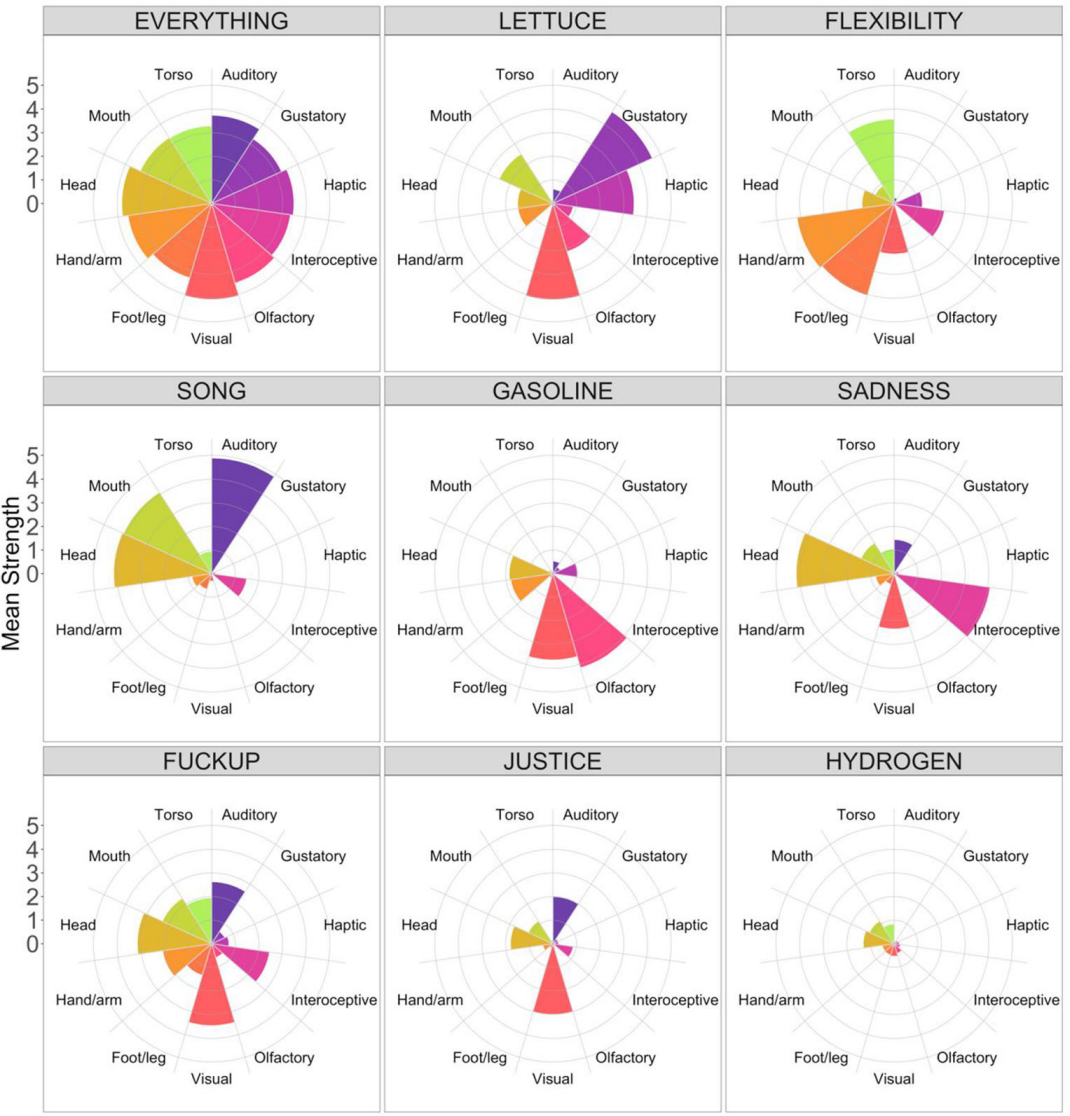
Polar plots for a range of individual concepts, showing the mean sensorimotor strength on each of the 11 dimensions. Similar plots can be automatically generated using the web application associated with the norms

In Fig. 4, we provide some examples of how the sensorimotor profiles of particular concepts varied across dimensions. To quantify the distinctness of the information captured by each dimension, we ran principal component analysis (PCA) across all 11 dimensions and examined the uniqueness scores per dimension (i.e., the proportion of variance from each dimension that is unique and not shared with any extracted components). A parallel analysis (95th percentile) determined that the optimal number of components to extract was four, and the resulting uniqueness scores are given in Table 2. Overall, all 11 dimensions captured distinct information to varying extents: 12.2% of the information in gustatory strength was unique, whereas 43.0% of the information in head action strength was unique, with all other dimensions positioned in between.

Following Lynott and Connell (2009, 2013), we also calculated and included additional variables of interest for each word (numbers of participants who knew each item, modality and effector exclusivity, dominant dimension). In the following sections of results, we discuss these variables before examining the interrelationships between dimensions. The Appendix contains a full list of all variables included in the norms.

#### Number and percentage known

We include in the norms a number of fields relating to how well-known each concept was in our sample of participants, calculated separately for the perceptual and action strength components. The number of participants who provided a valid rating for the concept rather than checking the “don’t know” box (N_known.perceptual, N_known.action) and the number of valid participants who completed the item list featuring the concept (List_N.perceptual, List_N.action) formed the basis of calculating the proportion (0–1) of participants who knew the concept (Percent_known.perceptual, Percent_known.action). We note that fewer participants knew the concepts in our sensorimotor strength norms (*M* = 92.8% for perceptual component, *M* = 94.3% for action component) than did the participants in Brysbaert et al.’s (2014) concreteness norms (*M* = 96.5%). Indeed, even though all concepts in our norms were known by at least 85% of Brysbaert et al.’s concreteness participants, a sizeable number of concepts were known by less than 85% of our participants: some 7,205 items in the perceptual component and 4,940 items in the action component. For instance, the item *languidly* was known by only 46% (16 out of 35 participants) in perceptual strength norming and by 51% (18 out of 35 participants) in action strength norming, in contrast to 90% in Brysbaert et al.’s concreteness norms. Since the participants in our sensorimotor norms and Brysbaert et al.’s concreteness norms were drawn from the same pool (i.e., MTurk workers), it is unlikely that sampling differences alone could have produced the divergence in what is considered “known.” Although sample-based differences may have been at play, we speculate that the reason for this difference is that rating sensorimotor strength on individual modalities or effectors requires a more specific, detailed conceptual representation—that is, a deeper understanding of what the word means—than rating concreteness. Although 90% of participants felt they knew the word *languidly* well enough to rate it as a slightly abstract concept, only around half of respondents felt their understanding of *languidly* was up to rating the details of its sensorimotor experience. The more detailed the required semantic processing, the more conservative people appear to become in deciding that they “know” a word.

#### Exclusivity scores

We calculated exclusivity scores per item (i.e., a measure of the extent to which a particular concept is experienced through a single dimension) as the rating range divided by the sum (Lynott & Connell, 2009). Exclusivity scores can be expressed as a proportion, and extend from 0 (completely multidimensional and experienced equally in all dimensions) to 1.0 (completely unidimensional and experienced solely through a single dimension). To allow for separate consideration of the perceptual and action components of the norms, we report in the norms separate exclusivity scores across the six perceptual modalities (i.e., Exclusivity.perceptual), the five action effectors (i.e., Exclusivity.action), and the 11 sensorimotor dimensions (i.e., Exclusivity.sensorimotor). Overall, the concepts were highly multidimensional, with a mean sensorimotor exclusivity score of 24.0% (*SD* = 6.53%), and tended to involve a wider range of action effectors (effector exclusivity *M* = 34.4%, *SD* = 15.2%) than perceptual modalities (modality exclusivity *M* = 43.5%, *SD* = 13.0%) in their representations. The mean modality exclusivity of the present norms for 39,707 concepts is very similar to values from previous work, lying between those found for a sample of 400 nouns (39.2%: Lynott & Connell, 2013) and 423 adjectives (46.1%: Lynott & Connell, 2009), even though the present norms include an additional perceptual modality of interoception.

By way of example, the most multidimensional word in the norms is *everything*, with sensorimotor exclusivity of 2.1%, which—perhaps unsurprisingly—is because *everything* is experienced relatively strongly across all perceptual modalities and action effectors (rating range from 3.22 to 4.06). The most unidimensional word in the norms is *monochromatically*, with sensorimotor exclusivity of 62.9%, which resulted from the concept scoring strongly on visual strength (3.92) but weakly on everything else. However, it’s important to note that high exclusivity does not necessarily mean high strength. If one considers modality exclusivity alone, *rainbow* is strongly visual (4.68), whereas *unbudgeted* is weakly visual (1.35), but they both score 100% on modality exclusivity (i.e., they are unimodally visual concepts) because all other modalities have perceptual strength of zero. Similarly, when it comes to effector exclusivity, *blink* strongly involves action with the head (4.75), whereas *shorebird* involves head action only weakly (0.71), but they both score 100% on effector exclusivity because all other effectors have action strength of zero. See Table 3 for the perceptual modality, action effector, and sensorimotor exclusivity scores per dominant dimension.

**Table 3:** Numbers of concepts per dominant perceptual modality, dominant action effector, and overall dominant sensorimotor dimension, with mean ratings of sensorimotor strength (0–5) and exclusivity scores (modality, effector, or sensorimotor)

Dominant Dimension	*N*	Sensorimotor Dimension											Exclusivity
		Auditory	Gustatory	Haptic	Intero-ceptive	Olfactory	Visual	Foot/ Leg	Hand/ Arm	Head	Mouth/ Throat	Torso	
*Perceptual Modality*													
Auditory	4,528	**3.04**	0.14	0.42	1.01	0.16	2.02	0.42	0.87	2.52	2.09	0.49	44.2%
Gustatory	890	0.50	**3.95**	1.69	0.91	2.36	2.96	0.23	1.53	1.75	3.51	0.67	29.5%
Haptic	975	0.82	0.36	**3.41**	1.24	0.36	2.68	1.21	2.76	1.80	0.98	1.25	37.4%
Interoceptive	3,546	1.34	0.33	0.73	**2.86**	0.34	1.88	0.85	1.11	2.67	1.54	1.20	36.8%
Olfactory	216	0.65	0.98	0.87	0.84	**3.58**	2.02	0.42	1.10	2.59	1.38	0.72	40.7%
Visual	29,552	1.36	0.24	1.12	0.81	0.35	**3.16**	0.87	1.53	2.22	1.04	0.81	44.8%
*Action Effector*													
Foot/leg	1,409	1.09	0.11	1.45	1.01	0.26	3.35	**2.91**	1.67	1.76	0.68	1.39	29.0%
Hand/arm	6,917	1.10	0.22	2.09	0.68	0.37	3.33	0.99	**2.74**	1.80	0.74	0.94	33.1%
Head	26,714	1.60	0.22	0.78	1.09	0.33	2.81	0.70	1.14	**2.49**	1.18	0.71	34.5%
Mouth/throat	3,707	2.04	1.36	0.97	1.08	0.88	2.53	0.39	1.15	2.00	**3.10**	0.67	38.9%
Torso	961	0.78	0.24	1.80	1.73	0.45	2.93	1.14	1.50	1.60	0.79	**2.75**	31.3%
*Sensorimotor Dimension*													
Auditory	2,690	**3.43**	0.13	0.45	0.93	0.15	2.15	0.40	0.89	2.37	2.08	0.47	25.5%
Gustatory	661	0.47	**4.11**	1.78	0.83	2.51	3.09	0.23	1.51	1.70	3.37	0.62	19.9%
Haptic	649	0.80	0.40	**3.57**	1.25	0.40	2.75	1.22	2.56	1.83	0.97	1.28	20.7%
Interoceptive	1,766	1.37	0.35	0.86	**3.26**	0.37	2.02	0.94	1.18	2.45	1.50	1.35	20.4%
Olfactory	176	0.59	1.03	0.86	0.81	**3.78**	2.12	0.42	1.07	2.49	1.31	0.74	25.1%
Visual	22,774	1.36	0.24	1.20	0.74	0.38	**3.36**	0.84	1.52	2.11	0.95	0.79	25.1%
Foot/leg	428	1.05	0.11	1.49	1.21	0.18	3.01	**3.66**	1.75	1.77	0.69	1.57	22.5%
Hand/arm	1,434	1.04	0.17	2.08	0.88	0.26	2.83	1.17	**3.46**	1.90	0.79	1.14	22.1%
Head	7,740	1.57	0.21	0.52	1.37	0.25	2.13	0.66	1.05	**2.90**	1.45	0.72	22.5%
Mouth/throat	1,166	1.87	1.17	0.88	1.30	0.70	2.23	0.41	1.18	2.14	**3.51**	0.78	21.5%
Torso	223	0.74	0.23	1.48	1.88	0.36	2.24	1.04	1.38	1.57	0.87	**3.17**	21.7%

The mean rating for each dominant dimension is in bold.

#### Dominant modalities and effectors

We identified the dominant dimension of each concept in the norms according to which dimension has the highest rating (i.e., maximum sensorimotor strength), and labeled the dominant perceptual modality, dominant action effector, and the overall dominant sensorimotor dimension per item. When there was a tie for the highest rating (perceptual, *N* = 593; action, *N* = 706; sensorimotor, *N* = 478), we assigned a dominant dimension at random from the tied candidates.^2^

Table 3 shows the numbers of concepts per dominant dimension as well as the mean ratings per dimension in each case; the means are shown separately according to whether the concept is classified by dominant perceptual modality, dominant action effector, or overall dominant sensorimotor dimension. For example, 4,528 words had auditory as the dominant perceptual modality, and based on just those words, the mean auditory rating was 3.04, whereas the mean gustatory rating for those words was 0.14. As had previously been found in small-scale perceptual strength norms (that focused either on highly perceptual concepts—Lynott & Connell, 2009; Winter, Perlman, & Majid, 2018—or a representative random selections of concepts—Lynott & Connell, 2013), vision dominates the perceptual modalities. Here we show that vision is the overall dominant sensorimotor dimension: More concepts are dominated by vision than by the other ten dimensions put together. Some 57% of words in English are visually dominant, and—since our item set of approximately 40,000 words was chosen to represent a full adult vocabulary—this means that the majority of English word meanings are grounded in visual experience. Considering perceptual modalities alone, vision is the most important modality (74% of items are visually dominant; e.g., *cloud*, *mirror*), followed by auditory (11%; *thunder*, *mockery*), interoceptive (9%; *ulcer*, *empathy*), haptic (2%; *prickly*, *blister*), gustatory (2%; *pizza*, *sweet*), and olfactory (< 1%; *perfume*, *skunk*). When considering action effectors alone, the most important effector is the head excluding mouth and throat; 67% of items are dominated by head action; e.g., *stare*, *daydream*), followed by the hand and arm (17%; *throw*, *fork*), mouth and throat (9%; *eat*, *pronounce*), foot and leg (4%; *kick*, *sandal*), and torso (2%; *slouch*, *polo shirt*).

#### Relationship between dimensions

**Fig. 5:**
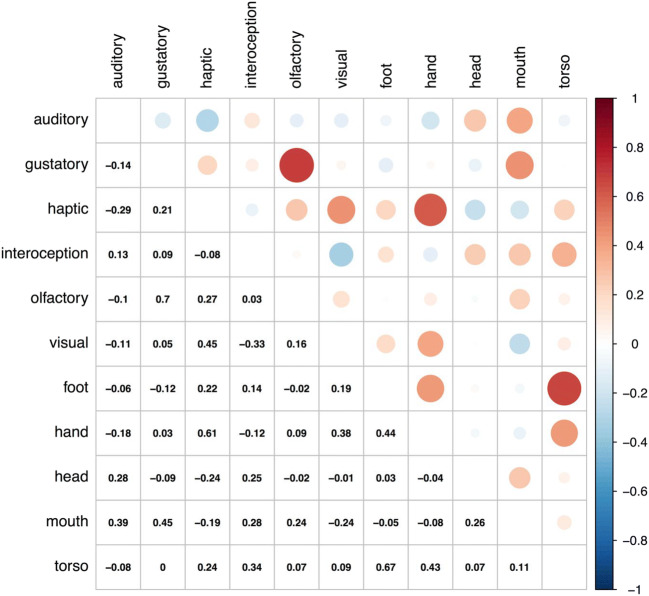
Correlation matrix plot between the 11 dimensions for mean ratings of sensorimotor strength (*N* = 39,707). Larger circles indicate stronger correlations, with red shades being positive and blue shades being negative

The correlations between individual perceptual modalities and action effectors are shown in Fig. 5, a correlation matrix plot between all 11 dimensions for the mean ratings of sensorimotor strength (*N* = 39,707). Given the exploratory nature of the analysis (i.e., without hypotheses to confirm), we opted not to include inferential statistics such as *p* values, and report only the correlation coefficient. As we had previously found for perceptual strength norms (Lynott & Connell, 2009, 2013; see also Connell, Lynott, & Banks, 2018), gustatory and olfactory strength were highly correlated (e.g., foodstuffs like *peach* are experienced via taste and smell), as were visual and haptic strength (e.g., most objects and textures that can be touched are also visible, such as *bowl* or *prickly*), whereas auditory strength was negatively related to all other modalities except interoception (e.g., an auditory experience such as *rhythm* or *beep* is often difficult to touch, taste, smell, or see). Interoception was also negatively correlated with vision, reflecting the fact that sensations inside the body (e.g., *nausea*, *heartbreak*) are typically not visible. Nonetheless, despite their intercorrelations, all modalities were distinct for at least some concepts. For example, some olfactory experience does not relate to food, and hence has no gustatory counterpart (e.g., *perfume*), and some taste concepts involve no smell (e.g., *salty*). Similarly, some haptic experience is not easily visible (e.g., *heat*, *clammy*), and many visually strong concepts cannot be touched (e.g., *cloud*, *yellow*).

Turning to the relationships between action effectors, we found that the strengths of foot and torso action were highly correlated. One likely explanation for the correlation between the foot and torso action ratings is that in the real world, many actions of the foot/leg also involve the torso, such as *sitting* and *climbing*, as do experiences of objects such as *bed*, *bath*, and *clothing*. To a slightly lesser extent, but for much the same reasons, hand action strength was also correlated with foot and torso action strength (e.g., *climbing* and *bath* also involve experience of hand action). Head and mouth action strength were also moderately correlated, plausibly because acts of social communication involve action with both the mouth/throat for vocalization and the rest of the head for attending and facial expression, such as *debate*, *storytelling*, and *laughing*. Nonetheless, mouth and head actions were separable: Food and eating concepts tend to involve strong mouth action but weak head action (e.g., *bagel*, *chew*). Moreover, experiences that involve strong head action but weak mouth action include those relating to hearing (e.g., *listening*, *noisy*, *smoke alarm*) and interoception (e.g., *earache*, *dizzy*, *delirium*).

Indeed, action effectors add a number of new insights to the relationship between perception and action in the representation of concepts. As we suggested above, the role of head action strength in hearing and interoceptive experiences underlies its correlation with auditory and interoceptive strength. Haptic perceptual strength is strongly correlated with experience of hand action (perhaps unsurprisingly), but also to some extent with foot and torso action: that is, people mainly feel through touch by using their hands, but also by using other major body parts. Visual strength also correlates with hand action, consistent with the visual-haptic relationship that most touchable things can be seen. Mouth action strength correlated negatively with visual and haptic strength (e.g., most things that are seen and touched are not placed in the mouth, such as *dog* and *prickly*), but positively with all other modalities. Mouth action and auditory strength were correlated mostly due to experience of vocalizations (e.g., *sing*, *poetry*, *roar*), and experiences of food and eating (e.g., *chocolate*, *sip*) accounted for the relationship between mouth action and gustatory/olfactory strength. Finally, mouth action also correlated with interoceptive strength, primarily for concepts that were identified by Connell, Lynott, and Banks (2018) as domains of interoceptive experience, such as (mal)function of the respiratory and digestive systems (e.g., *vomiting*, *breathe*, *sneezing*) and narcotics (e.g., *amphetamine*, *tequila*). Similarly, torso action correlates with interoceptive strength for concepts relating to digestion (e.g., *gastrointestinal*, *hungry*) and (ill) health (e.g., *flu*, *heart attack*, *achiness*).

**Fig. 6:**
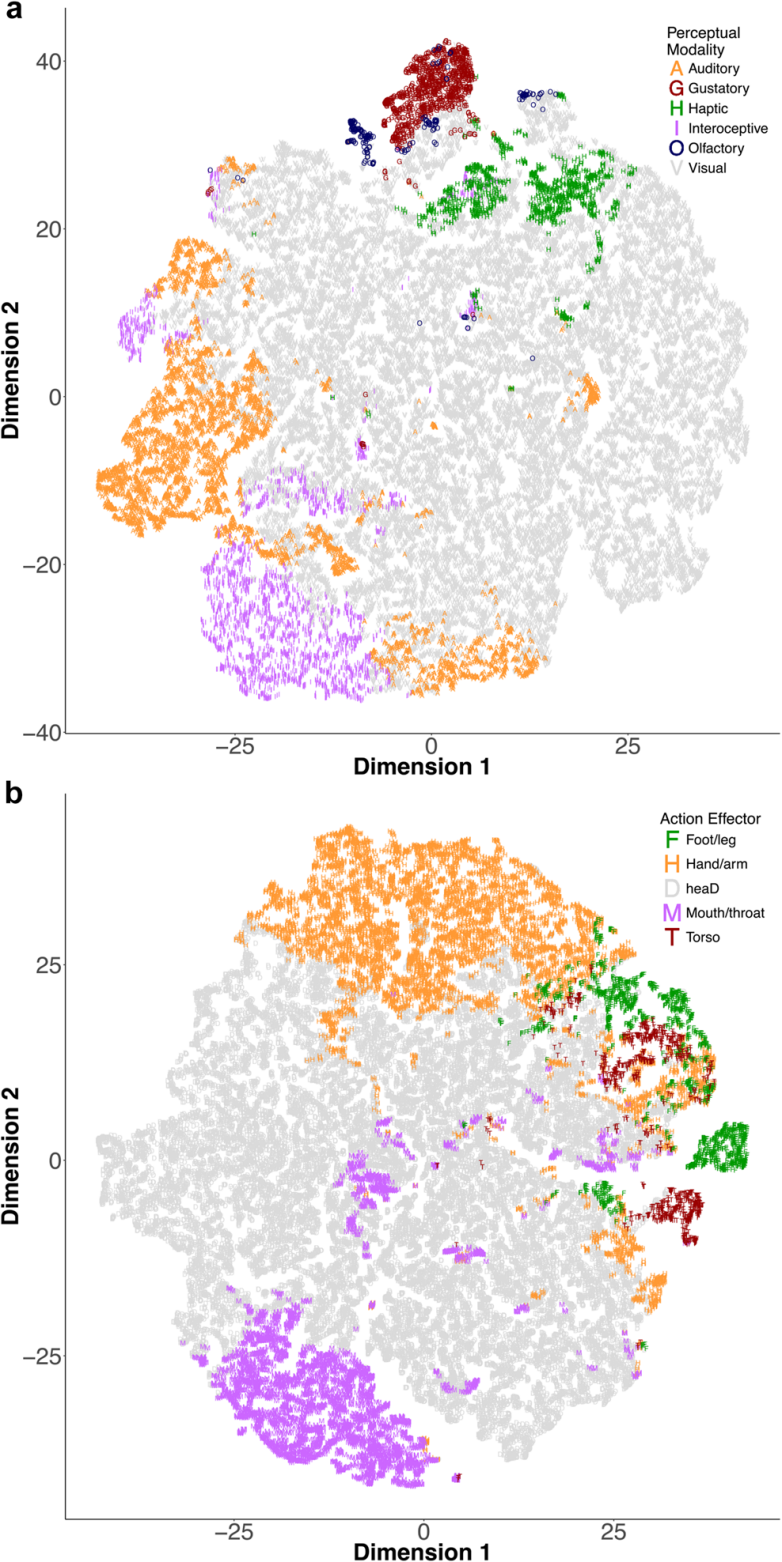
The *t*-distributed stochastic neighbor embedding (*t*-SNE) plots of the perceptual strength norms (panel A, labeled by the dominant modality) and the action strength norms (panel B, labeled by the dominant effector). The plots provide two-dimensional visualizations of the continuity between concepts that are rated highly in the same dimensions

The relationships between individual perceptual modalities and action effectors are also illustrated in the continuity between concepts that are rated highly in the same dimensions (see Fig. 6, which contains separate *t*-SNE plots for the perceptual and action components, labeled by dominant modality/effector^3^). As we noted earlier, vision dominates the perceptual modalities, and head action dominates the effectors, but other dimensions are nonetheless distinct.

## Study 2a: Identifying the optimal composite variable of sensorimotor experience

Our goals in this validation study were twofold: we wished to empirically determine the best composite strength variable (i.e., single value) for representing a concept’s multidimensional sensorimotor profile, and to demonstrate the utility of sensorimotor strength as a semantic predictor in word processing. Although an 11-dimension sensorimotor profile is a rich source of semantic information about a particular concept, it can nonetheless be somewhat unwieldy for some uses. It is often valuable to aggregate multiple dimensions into a single composite variable, such as for use as a predictor in regression analyses without unnecessarily inflating the number of parameters, or for fair comparison with other single-variable semantic measures. There are many different potential methods of creating such a composite variable. Previous work on perceptual strength had used the strength of the dominant modality (i.e., the maximum perceptual strength rating across all modalities) as the preferred composite variable (e.g., Connell & Lynott, 2016a; Connell et al., 2018; Winter et al., 2018), finding that it offered a better fit to visual word recognition performance than did alternative measures (Connell & Lynott, 2012a). However, work in Serbian (Filipović Đurđević et al., 2016) showed that the best fit emerged from summed perceptual strength (i.e., sum of perceptual strength ratings across all modalities) or vector length (i.e., Euclidean distance of the multidimensional vector of perceptual strength ratings from the origin). It is difficult to be certain whether this variability is due to language differences (i.e., English vs. Serbian) or to sampling differences (i.e., hundreds of words with limited overlap). We therefore sought to determine the best single composite variable for our 11-dimension sensorimotor norms by using a much larger and more representative sample of concepts in English (i.e., tens of thousands of words). As in previous studies (e.g., Connell & Lynott, 2012a), we judged the “best” variable to be the one that offers the best fit to lexical decision latency, a task in which semantic facilitation emerges from automatic and implicit access to the sensorimotor basis of the concept (i.e., the sensorimotor simulation of word meaning). To ensure the generalizability of the best composite variable and demonstrate its utility as a semantic predictor in visual word recognition, we examined lexical decision performance across two different datasets (i.e., English Lexicon Project, British Lexicon Project) and using two performance measures (response time and accuracy).

### Method

#### Materials

We collated a set of 22,297 words that represented the intersection of data between our sensorimotor strength norms and lexical decision data from the English Lexicon Project (ELP; Balota, et al., 2007), and a separate set of 11,768 words that represented the intersection with the British Lexicon Project (BLP; Keuleers, Lacey, Rastle, & Brysbaert, 2012). For each ELP and BLP dataset, we extracted the dependent variables of zRT (i.e., lexical decision RT standardized per participant) and accuracy, as well as a set of lexical predictors that typically predict lexical decision performance (log SUBTLEXus word frequency, length in letters, number of syllables, and orthographic Levenshtein distance to the 20 nearest neighbors).

#### Candidate composite variables

There are a number of ways to reduce an 11-dimension profile of sensorimotor strength to a single composite variable, and we examined the six most promising candidates. Most of the candidate variables we tested are distance metrics in vector space of a particular concept (i.e., an 11-dimension vector) from the origin. Minkowski distance (with exponent parameter *m*) is a generalization of these distance metrics: roughly speaking, the highest-value dimension always contributes to the calculated distance, and *m* determines the extent to which the other dimensions contribute according to how close their values are to the highest-value dimension (see, e.g., Han, Kamber, & Pei, 2011, p. 72). That is, low-value *m* means that all dimensions make noticeable contributions to the calculated distance, whereas high-value *m* means only the highest-value dimension(s) make noticeable contributions to the calculated distance.

Our set of candidate variables is as follows:

*Maximum strength*: the highest rating across the 11 sensorimotor dimensions for a concept. Theoretically, maximum strength represents sensorimotor strength of the dominant dimension, which has previously been found to be the best composite variable of perceptual strength (Connell & Lynott, 2012a, 2016a; Connell et al., 2018). Maximum strength is consistent with Chebyshev distance of the vector from the origin (Minkowski distance *m* = ∞), where the distance between two vectors is equal to the greatest of their differences along any coordinate dimension (Abello, Pardalos, & Resende, 2013).

*Minkowski 10 distance*: Minkowski distance at *m* = 10 of the vector from the origin. Theoretically, it represents the sensorimotor strength of the dominant dimension, plus an attenuated influence of any other dimensions that are nearly as strong as the dominant dimension.

*Minkowski 3 distance*: Minkowski distance at *m* = 3 of the vector from the origin. Theoretically, it represents the sensorimotor strength in all dimensions, but the influence of weaker dimensions is attenuated, and it has been proposed as the optimal value for modeling multisensory cue integration in perception (To, Baddeley, Troscianko, & Tolhurst, 2011). As compared to Minkowski 10, the Minkowski 3 distance receives a greater contribution from weaker dimensions.

*Euclidean vector length*: the straight-line distance of the vector from the origin (Minkowski distance *m* = 2). Theoretically, it represents the sensorimotor strength in all dimensions without attenuation, but the highest-value dimensions are most important, because they are farthest from the origin.

*Summed strength*: the sum of all ratings across the 11 sensorimotor dimensions for a concept, consistent with the Manhattan (i.e., city block) distance of the vector from the origin (Minkowski distance *m* = 1), which is statistically equivalent to mean strength as a predictor in regression analyses. Theoretically, it represents all dimensions with equal importance.

*PCA component*: the dimensionality reduction of all 11 dimensions via PCA to a single component (saved via regression method), with the computed factor standardized to a mean of zero. Theoretically, the PCA method preserves the common variance of all 11 dimensions (24.8% of overall original variance; 34.4% of the variance of the perceptual strength norms; 34.4%, and 40.6% of the variance of the action strength norms) in a single variable.

#### Design and analysis

We performed Bayesian linear regressions on four dependent variables—zRT and accuracy from both ELP and BLP—using JSZ default priors (*r* scale = .354) and Bayesian adaptive sampling (JASP Team, 2019). To reproduce this analysis, JASP analysis files are deposited in the OSF repository. In each analysis, we built a null model of lexical predictors (log SUBTLEX word frequency, number of letters, number of syllables, orthographic Levenshtein distance), and then added a single candidate composite variable to form the alternative model and used Bayes factors (BF_10_) to quantify the evidence in favor of the alternative model over the null. Finally, we compared BFs across models to select the best-performing composite variable for each dependent variable. Due to the magnitude of the BF values, we report natural log BFs throughout.

### Results and discussion

Overall, we found that all composite variables of the sensorimotor norms reliably predicted lexical decision performance above and beyond the null model of lexical predictors, for both response times and accuracy, in both the ELP and BLP datasets. In Table 4, we report the log BF per candidate composite variable, as well as proportion of variance explained by each variable (i.e., *R*^2^ change from the null model). The best-performing composite variable of sensorimotor strength was Minkowski 3 strength, which consistently outperformed the other candidates across all four dependent measures (i.e., RT and accuracy in both ELP and BLP). Although several candidate variables explained similar amounts of variance in lexical decision performance (e.g., Minkowski 10, Minkowski 3, and Euclidean distance all explain 0.7% of the variation in ELP RTs), BFs clearly differentiated between their strength of evidence. Model comparisons showed that the data were between a thousand and several million times more likely under a model with Minkowski 3 strength than under the next-best candidate variable.^4^

**Table 4:** Bayesian linear regression results for English Lexicon Project (ELP) and British Lexicon Project (BLP) lexical decision response times (RTs) and accuracy, showing log Bayes factors (logBF_10_) for each candidate composite variable of sensorimotor strength against the null model

Dependent Measure	Model	**English Lexicon Project (*N*= 22,297)**			**British Lexicon Project (*N*= 11,768)**		
		logBF_10_	*R*^2^	**∆***R*^2^	logBF_10_	*R*^2^	**∆***R*^2^
RT	*Null* (*lexical variables)*		*.591*			*.485*	
	Max strength	165.420	.597	.006	175.912	.501	.016
	Minkowski 10	177.86	.598	.007	193.544	.502	.017
	**Minkowski 3**	**202.551**	**.598**	**.007**	**228.285**	**.505**	**.020**
	Euclidean	192.129	.598	.007	210.165	.503	.018
	Summed strength	142.813	.596	.005	136.651	.497	.012
	PCA	54.532	.593	.002	50.747	.490	.005
Accuracy	*Null* (*lexical variables)*		*.237*			*.286*	
	Max strength	88.240	.244	.007	105.252	.299	.013
	Minkowski 10	93.828	.244	.007	114.398	.300	.014
	**Minkowski 3**	**102.067**	**.245**	**.008**	**138.688**	**.303**	**.017**
	Euclidean	93.375	.244	.007	131.714	.302	.016
	Summed strength	69.975	.242	.005	92.785	.298	.012
	PCA	43.377	.241	.004	29.613	.290	.004

Minkowski 3 strength therefore represents an optimal means of aggregating 11 dimensions of sensorimotor strength into a single composite variable. It acted as a powerful semantic facilitator in lexical decision performance, explaining 0.7%–0.8% of variance in the ELP dataset, and 1.7%–2.0% of variance in the BLP dataset (above the null model of lexical predictors), which compares favorably with other semantic facilitation effects in the literature (e.g., Connell & Lynott, 2012a, 2016a; Kuperman et al., 2014). The previous best composite variable for perceptual strength, maximum strength in the dominant dimension, also performed very well, demonstrating that the dominant perceptual modality or action effector is indeed highly important for semantic facilitation in visual word recognition. Nonetheless, the superior performance of Minkowski 3 strength indicates that other sensorimotor dimensions that are strongly involved in a concept’s experience—even if they are not dominant—are also important for semantic facilitation. We return to the importance of the Minkowski 3 measure in the General Discussion.

## Study 2b: Validating the independent utility of perceptual and action strength

Identifying the optimal composite variable of Minkowski 3 strength leads us to our second goal: We wished to replicate the utility of perceptual strength ratings in modeling people’s performance in cognitive tasks, and establish the independent utility of action strength as a performance predictor. Although perceptual strength has enjoyed a number of empirical demonstrations of its effect on behavior, in tasks such as word recognition and modality detection (e.g., Connell & Lynott, 2010, 2012a, 2016a; Connell et al., 2018), the same is not true of action strength. Previous work has shown that other action-related variables can predict word recognition performance (Siakaluk, Pexman, Aguilera, Owen, & Sears, 2008) and semantic judgments (Pexman, Muraki, Sidhu, Siakaluk & Yap, 2019), although no study to date has examined the effect of action experience using the range of effectors and the scale of the present sensorimotor norms. It is therefore important to evaluate our particular measures of action strength in their own right. Specifically, it is important to establish how the effects of our chosen composite sensorimotor strength variable may be due to both perceptual and action dimensions. In our final validation exercise, we therefore calculated our optimal composite variable separately for perceptual and action strength (i.e., Minkowski 3 distance calculated separately for six dimensions of perceptual strength and five dimensions of action strength) and examined their relative contributions in predicting lexical decision performance.

### Method

We utilized the same datasets as in Study 2a, and the method was the same as in Study 2a, with the following exceptions.

#### Composite variables

Rather than calculate our composite variables across all 11 dimensions of sensorimotor strength, we calculated Minkowski 3 perceptual strength (i.e., Minkowski distance at *m* = 3 of the six-dimension perceptual strength vector from the origin) and Minkowski 3 action strength (i.e., Minkowski distance at *m* = 3 of the five-dimension action strength vector from the origin).

#### Design and analysis

Bayesian linear regressions were, as per Study 2a (using JSZ default priors, *r* scale = .354, with Bayesian adaptive sampling; JASP Team, 2019), but with different model comparisons. As per previous studies reported above, all data and analysis scripts are downloadable from the project’s OSF page. Specifically, we created models to examine the effect of Minkowski 3 perceptual strength (Model 1), Minkowski 3 action strength (Model 2), and Minkowski 3 perceptual strength + Minkowski 3 action strength simultaneously (Model 3), and quantified their evidence relative to the null model (containing the same lexical predictors as above) using Bayes factors. We then compared the relative evidence for action strength and perceptual strength as individual predictors (Model 2 vs. Model 1), and for the ability of action strength to predict lexical decision above and beyond perceptual strength (Model 3 vs. Model 1).

### Results and discussion

Both the perceptual and action strength Minkowski 3 composite variables independently predicted lexical decision performance across both the ELP and BLP datasets (see Table 5). Replicating previous findings (e.g., Connell & Lynott, 2010, 2012a, 2016a; Connell et al., 2018), perceptual strength accounted for 0.5%–1.6% of the variance in RTs and 0.5%–1.2% of the variance in accuracy. As an independent predictor, the effect size of action strength was overall fairly similar to that of perceptual strength: 0.6%–1.2% of variance in RTs, and 0.6%–1.3% of variance in accuracy. In three of the four dependent measures (ELP RT and accuracy, BLP accuracy), the effect of action strength was stronger than that of perceptual strength; only in the final dependent measure (BLP RT) was the effect of action strength weaker than that of perceptual strength. Combined entry of perceptual and action strength performed best, explaining 0.8%–2.0% of variance in RTs and 0.8%–1.8% of variance in accuracy. Moreover, since this combination of perceptual and action strength performed better than perceptual strength alone in all cases, this means that action strength explained unique variance in lexical decision performance above and beyond perceptual strength.

**Table 5:** Bayesian linear regression results for English Lexicon Project (ELP) and British Lexicon Project (BLP) lexical decision response times (RTs) and accuracy, showing log Bayes factors (logBF) for model comparisons with composite Minkowski 3 perceptual strength and Minkowski 3 action strength

Dependent Measure	Model Comparison	**English Lexicon Project (*N*= 22,297)**			**British Lexicon Project (*N*= 11,768)**		
		logBF	*R*^2^	**∆***R*^2^	logBF	*R*^2^	**∆***R*^2^
RT	*Null* (*lexical variables)*		*.591*			*.485*	
	Perceptual strength vs. null (BF_10_)	141.337	.596	.005	182.839	.501	.016
	Action strength vs. null (BF_20_)	149.563	.597	.006	138.873	.497	.012
	Perceptual + action strength vs. null (BF_30_)	207.094	.599	.008	230.205	.505	.200
	Action vs. perceptual strength (BF_21_)	8.226		.001	– 43.966		– .004
	Perceptual + action strength vs. perceptual strength only (BF_31_)	65.757		.003	47.366		.004
Accuracy	*Null* (*lexical variables)*		*.237*			*.286*	
	Perceptual strength vs. null (BF_10_)	66.951	.242	.005	98.251	.298	.012
	Action strength vs. null (BF_20_)	81.216	.243	.006	105.771	.299	.013
	Perceptual + action strength vs. null (BF_30_)	104.765	.245	.008	7.520	.304	.018
	Action vs. perceptual strength (BF_21_)	14.265		.001	7.520		.001
	Perceptual + action strength vs. perceptual strength only (BF_31_)	37.814		.003	46.023		.006

Overall, these findings clearly demonstrate the continued utility of perceptual strength and the novel utility of action strength as predictors of lexical decision performance. We note that the two-parameter model of perceptual + action strength explained amounts of variance similar to that explained by the single-parameter model of sensorimotor strength in Study 2a. Nonetheless, there may be occasions when it is theoretically important to separate semantic information of a perceptual origin from that of an action origin. We therefore provide in the norms the overall composite variable of Minkowski 3 sensorimotor strength, as well as separate variables for Minkowski 3 perceptual strength and Minkowski 3 action strength.

## General discussion

The Lancaster Sensorimotor Norms present validated ratings of sensorimotor strength for almost 40,000 words. These ratings span 11 distinct dimensions of sensorimotor experience: six perceptual modalities (auditory, gustatory, haptic, interoceptive, olfactory, visual) and five action effectors (foot/leg, hand/arm, head, mouth, torso). The norms also include a further 12 cross-dimensional variables (e.g., exclusivity, dominant dimension, maximum strength, and the optimal composite variable of Minkowski 3 strength) for overall sensorimotor strength as well as for the perceptual and action components individually, plus information about what proportion of participants know each word well enough to rate its perceptual or action strength. The norms show good reliability, with strong levels of agreement across all dimensions, and their utility is demonstrated in capturing lexical decision behavior from two different databases. Altogether, the Lancaster Sensorimotor Norms provide over one million informative data points regarding the sensorimotor basis of semantics and conceptual grounding, and represent the largest set of psycholinguistic norms in English to date.

In comparison to previous norms, the Lancaster Sensorimotor Norms offer a number of advances. First is the sheer scale of the norms in terms of both lexical coverage and number of dimensions. Most semantic norms are available for only a few hundred (e.g., Amsel et al., 2012; Binder et al., 2016; Lynott & Connell, 2009, 2013; Paivio, Yuille, & Madigan, 1968) or a few thousand words/concepts (e.g., Cortese & Fugett, 2004; Juhasz & Yap, 2013; Scott, Keitel, Becirspahic, Yao, & Sereno, 2019; Tillotson et al., 2008). Indeed, there is generally an inverse relationship between the number of lexical items and the number of semantic variables normed, where multidimensional norms tend to have small coverage (e.g., Binder et al., 2016, provide ratings for 65 dimensions on 535 concepts), whereas high-coverage norms tend to be for single dimensions (e.g., Brysbaert et al., 2014, provide ratings of concreteness for 39,954 concepts). By contrast, we provide norms for 11 sensorimotor dimensions (plus cross-dimensional variables) for 39,707 concepts, which is large enough to represent a full-sized adult vocabulary;^5^ and comprehensive enough to span all perceptual modalities and action effectors involved in sensorimotor experience.

A second advance is that the norms provide a very rich source of information regarding the sensorimotor basis of semantics and conceptual grounding, and include novel dimensions of interoceptive strength, torso action strength, and separable mouth action and head action strength that have not previously been normed. As well as replicating previous findings on a larger scale, such as the dominance of visual vocabulary in English (Lynott & Connell, 2009, 2013; see also Winter et al., 2018) and the strong interrelationships between vision and touch and between taste and smell (Louwerse & Connell, 2011; Lynott & Connell, 2009, 2013), the breadth of dimensions also generates new insights into the sensorimotor basis of concepts. For example, interoceptive strength is highly important to abstract, particularly emotional, concepts (Connell et al., 2018). Or action verbs such as *run* or *carry*, that might seem to relate only to the foot/leg or hand/arm, often involve a moderate amount of torso action. Moreover, the present norms also offer important new insights regarding relationships between sensorimotor dimensions and their role in different concept types. For instance, auditory strength is negatively related to all dimensions except for head and mouth strength, where their positive relationship is primarily due to the importance of listening in speech and communication. By contrast, the strength of torso action is positively related to all dimensions except auditory and gustatory strength, highlighting its relevance to all but sound-related and taste-related concepts.

Finally, we identified a new composite variable that aggregates the information from the full 11-dimension sensorimotor profile in an optimal way to predict implicit access to semantic information: Minkowski 3 strength. In previous uses of perceptual strength norms across hundreds of items (Connell & Lynott, 2012a), we identified maximum perceptual strength (i.e., the strength in the dominant dimension) as the best single aggregate variable in predicting lexical decision performance (Study 2a). Although max strength continued to perform very well in the present analyses of tens of thousands of items, it was outperformed by the Minkowski 3 measure: a vector-based distance metric that allows nondominant dimensions to have an attenuated influence on the final score, according to how close their values are to that of the dominant dimension. It is notable that Minkowski 3 emerged as the best aggregate predictor of sensorimotor semantic facilitation, as Minkowski values around 3 have previously been identified as the optimal model for integrating multiple perceptual cues (To et al., 2011). Although Minkowski values in the range of 3–4 have previously been used in studies of visual perception (Quick, 1974; Robson & Graham, 1981), To and colleagues provided evidence that Minkowski values between 2.5 and 3 actually represent a general principle for perceptual integration and may reflect the summation of neural responses to perceptual stimuli. From the perspective of the present norms, it should be noted, of course, that one loses much information by trying to reduce a multidimensional representation to a single value, and considering individual perceptual modalities and action effectors is critical to a variety of theoretical questions (e.g., Connell & Lynott, 2014a; Connell et al., 2018; Winter et al., 2018). Nonetheless, Minkowski 3 strength provides a powerful tool when the necessity arises.

In terms of processing and representing concepts, what does it mean that Minkowski 3 outperforms other measures in predicting lexical processing behavior? Firstly, it suggests that in modeling human performance we need to take into account multiple sensorimotor dimensions, and that reliance on a single, dominant dimension of perception or action experience is inadequate for capturing performance. Second, it suggests that although all dimensions are important to some extent, not all dimensions should be treated equally. In other words, greater weighting should be placed on higher-value dimensions (i.e., those for which sensorimotor experience is strongest), but weaker dimensions should still be taken into account. If an equal weighting of all dimensions were the best approach, then the “summed strength” measure would have emerged as a better predictor in Study 2a. Similarly, if the best approach were to ignore or severely attenuate weaker dimensions, then measures such as maximum strength or Minkowski 10 would have emerged as better predictors. Finding that Minkowski 3, in its weighted balance of strong and weak dimensions, is a good predictor of lexical-processing performance may provide some insights for those interested in the sensorimotor correspondence of word meaning. For example, it may suggest a starting point for computational models in how best to combine signals from multiple sensorimotor regions of the brain to create composite semantic neural representations, that approximate the neural patterns observed in humans. As well as applying elements such as Minkowski 3 weightings to sensorimotor representations, there may be other ways in which the present work can act as a constraint on future theory development. For example, an understanding of the complex correlations between perceptual modalities and action effectors may constrain accounts of how attention may be implicitly allocated, switched, or shared in order to make optimal use of neural resources to meet task demands (e.g., Connell & Lynott, 2010, 2014a; Spence, Nicholls, & Driver, 2001). To make progress in this area, future theories will need to account for observed modality-specific effects, and cross-modal or cross-effector correspondences that impact how we process language and indeed the world around us.

In conclusion, the Lancaster Sensorimotor Norms provide a valuable resource for researchers across a variety of fields. Use of these norms as semantic variables, whether as an aggregate measure of sensorimotor strength or as modality- and effector-specific ratings, will inform psycholinguistic models of word recognition and language processing (e.g., Connell & Lynott, 2016a; Estes, Verges, & Adelman, 2015; Speed & Majid, 2017; see also Connell & Lynott, 2016b, for a review). The parallels between the sensorimotor dimensions in the present norms and specific brain regions related to perceptual and action processing mean that close examination of the roles and interactions of each dimension will be able to inform theories of grounded representation and embodied semantics (e.g., Connell et al., 2018; Lievers & Winter, 2018; Rey, Riou, Vallet, & Versace, 2017). For instance, we are currently using the norms to examine emergent conceptual structure from sensorimotor knowledge (Connell, Brand, Carney, Brysbaert, Banks, & Lynott, 2019; Connell, Brand, Carney, Brysbaert, & Lynott, 2019), and the role of sensorimotor experience in categorization (Banks, Wingfield, & Connell, 2019; van Hoef, Connell, & Lynott, 2019). Furthermore, the large size of the norms makes them amenable to some machine learning applications, such as using sensory language to identify early markers of clinical conditions (e.g., Kernot, Bossomaier, & Bradbury, 2017) or to inform recommender algorithms of therapeutic texts (Carney & Robertson, 2019). We hope the work presented here will prove useful for any researchers interested in the sensorimotor basis of word meaning and concepts.

### Open Practices Statement

The Lancaster Sensorimotor Norms dataset, additional data, materials, analysis, and scripts for all studies are available at https://osf.io/7emr6/. The norms dataset is also available as a searchable database (https://www.lancaster.ac.uk/psychology/lsnorms/).

### Author Note

This research was supported by the Leverhulme Trust, UK (Grant RPG-2015-412 to D.L., L.C., and M.B.) and the European Research Council (ERC) under the European Union’s Horizon 2020 research and innovation program, under Grant Agreement No. 682848 to L.C. We thank R. Meers and P. Mei, who provided help with data collection and quality control during the project, and D. Balota, for permitting us to include data from the English Lexicon Project in our deposited analysis files. We also thank B. Winter and two anonymous reviewers for helpful comments and feedback on our initial submissions.

### Open access note

All images and data used in this article are licensed under a Creative Commons Attribution 4.0 International License (CC-BY), which permits use, sharing, adaptation, distribution, and reproduction in any medium or format, as long as you give appropriate credit to the original author(s) and the source, provide a link to the Creative Commons license, and indicate if changes were made. Any images or other third-party material in this article are included in the article’s Creative Commons license, unless indicated otherwise in a credit line to the material. If material is not included in the article’s Creative Commons license and your intended use is not permitted by statutory regulation or exceeds the permitted use, you will need to obtain permission directly from the copyright holder. To view a copy of this license, visit http://creativecommons.org/licenses/by/4.0/.

## Appendix

Below are the variables included in the Lancaster Sensorimotor Norms dataset.

*Word*: Lemma (concept) presented to participants for rating

*Auditory.mean*: Auditory strength: mean rating (0–5) of how strongly the concept is experienced by hearing

*Gustatory.mean:* Gustatory strength: mean rating (0–5) of how strongly the concept is experienced by tasting

*Haptic.mean:* Haptic strength: mean rating (0–5) of how strongly the concept is experienced by feeling through touch

*Interoceptive.mean:* Interoceptive strength: mean rating (0–5) of how strongly the concept is experienced by sensations inside the body

*Olfactory.mean:* Olfactory strength: mean rating (0–5) of how strongly the concept is experienced by smelling

*Visual.mean:* Visual strength: mean rating (0–5) of how strongly the concept is experienced by seeing

*Foot_leg.mean:* Foot action strength: mean rating (0–5) of how strongly the concept is experienced by performing an action with the foot/leg

*Hand_arm.mean:* Hand action strength: mean rating (0–5) of how strongly the concept is experienced by performing an action with the hand/arm

*Head.mean:* Head action strength: mean rating (0–5) of how strongly the concept is experienced by performing an action with the head excluding mouth

*Mouth.mean:* Mouth action strength: mean rating (0–5) of how strongly the concept is experienced by performing an action with the mouth/throat

*Torso.mean:* Torso action strength: mean rating (0–5) of how strongly the concept is experienced by performing an action with the torso

*Auditory.SD:* Standard deviation of auditory strength ratings

*Gustatory.SD:* Standard deviation of gustatory strength ratings

*Haptic.SD:* Standard deviation of haptic strength ratings

*Interoceptive.SD:* Standard deviation of interoceptive strength ratings

*Olfactory.SD:* Standard deviation of olfactory strength ratings

*Visual.SD:* Standard deviation of visual strength ratings

*Foot_leg.SD:* Standard deviation of foot action strength ratings

*Hand_arm.SD:* Standard deviation of hand action strength ratings

*Head.SD:* Standard deviation of head action strength ratings

*Mouth.SD:* Standard deviation of mouth action strength ratings

*Torso.SD:* Standard deviation of torso action strength ratings

*Max_strength.perceptual:* Perceptual strength in the dominant modality (i.e., highest strength rating across six perceptual modalities)

*Minkowski3.perceptual:* Aggregated perceptual strength in all modalities in which the influence of weaker modalities is attenuated, calculated as Minkowski distance (with exponent 3) of the six-dimension vector of perceptual strength from the origin.

*Exclusivity.perceptual:* Modality exclusivity of the concept; the extent to which a concept is experienced though a single perceptual modality (0–1, typically expressed as a percentage), calculated as the range of perceptual strength values divided by their sum

*Dominant.perceptual:* Perceptual modality through which the concept is experienced most strongly (i.e., with highest perceptual strength rating)

*Max_strength.action:* Action strength in the dominant effector (i.e., highest strength rating across five action effectors)

*Minkowski3.action:* Aggregated action strength in all effectors in which the influence of weaker effectors is attenuated, calculated as Minkowski distance (with exponent 3) of the five-dimension vector of action strength from the origin.

*Exclusivity.action:* Effector exclusivity of the concept; the extent to which a concept is experienced though a single action effector (0–1, typically expressed as a percentage), calculated as the range of action strength values divided by their sum

*Dominant.action:* Action effector through which the concept is experienced most strongly (i.e., with highest action strength rating)

*Max_strength.sensorimotor:* Sensorimotor strength in the dominant dimension (i.e., highest strength rating across 11 sensorimotor dimensions)

*Minkowski3.sensorimotor:* Aggregated sensorimotor strength in all dimensions where the influence of weaker dimensions is attenuated, calculated as Minkowski distance (with exponent 3) of the 11-dimension vector of sensorimotor strength from the origin.

*Exclusivity.sensorimotor:* Sensorimotor exclusivity of the concept; the extent to which a concept is experienced though a single sensorimotor dimension (0–1, typically expressed as a percentage), calculated as the range of sensorimotor strength values divided by their sum

*Dominant.sensorimotor:* Sensorimotor dimension through which the concept is experienced most strongly (i.e., with highest sensorimotor strength rating)

*N_known.perceptual:* Number of participants in perceptual strength norming who knew the concept well enough to provide valid ratings (i.e., as opposed to selecting the “don’t know” option)

*List_*N.*perceptual:* Number of valid participants in perceptual strength norming who completed the item list featuring the concept (i.e., who were presented with the concept for rating)

*Percent_known.perceptual:* Percentage of participants (0–1) in perceptual strength norming who knew the concept well enough to provide valid ratings, calculated as N_known.perceptual divided by List_N.perceptual

*N_known.action:* Number of participants in action strength norming who knew the concept well enough to provide valid ratings (i.e., as opposed to selecting the “don’t know” option)

*List_*N.*action:* Number of valid participants in action strength norming who completed the item list featuring the concept (i.e., who were presented with the concept for rating)

*Percent_known.action:* Percentage of participants (0–1) in action strength norming who knew the concept well enough to provide valid ratings, calculated as N_known.action divided by List_N.action

*Mean_age.perceptual:* Average age of participants in perceptual strength norming who completed the item list featuring the concept (i.e., who were presented with the concept for rating)

*Mean_age.action:* Average age of participants in action strength norming who completed the item list featuring the concept (i.e., who were presented with the concept for rating)

*List#.perceptual:* Code number of the item list in perceptual strength norming that featured the concept

*List#.action:* Code number of the item list in action strength norming that featured the concept

## Footnotes

1 We discovered after norming was complete that two of Brysbaert et al.’s (2014) items had been rendered incorrectly in our item set, due to file reading errors: *infinity* had been rendered as *inf*, and *null* had been rendered as *nan*. We opted to retain *inf* and *nan* in our norms because they are words in their own right (i.e., *inf* is a common coding term to represent the value of infinity, and *nan* is a shortened form of grandmother/granny), and because they had been rated by participants consistently enough to pass our data-checking and exclusion criteria.

2 We applied this method for consistency with the existing norms, but others may prefer to apply alternative methods of distinguishing between tied cases.

3 The *t*-distributed stochastic neighbor embedding (*t*-SNE; Maaten & Hinton, 2008) approach is a nonlinear, probabilistic technique for dimensionality reduction that is well-suited to visualizing patterns in multidimensional data. In particular, the approach has been found to be good at preserving local structure from high-dimensional data sets in two-dimensional representations while also conveying global structure, and thus produces a visual display that best preserves the interdimensional relationships between items.

4 For ELP RTs, the evidence for Minkowski 3 strength was log BF = 10.42 times better than that for the next-best variable, Euclidean distance; for BLP RTs, Minkowski 3 was log BF = 18.12 times better than Euclidean distance; for ELP accuracy, Minkowski 3 was log BF = 8.24 times better than Minkowski 10; and for BLP accuracy, Minkowski 3 was log BF = 6.97 times better than Euclidean distance.

5 According to estimates by Brysbaert, Stevens, Mandera, and Keuleers (2016), typical adult native speakers of English in their mid-30s (i.e., the average age of the participants in our norms) would know approximately 46,000 lemmas, at a shallow level of knowing that a word exists. A subset of these lemmas would be known at a deeper level that involves some understanding of what the word means. Our norms for 39,707 lemmas required knowledge at a relatively deep level in order to rate the perceptual or action experience of the referent concept, and so provide reasonably generous coverage of a full-sized adult vocabulary.
